# INTRAMUSCULAR MARTIN-GRUBER ANASTOMOSIS

**DOI:** 10.1590/1413-785220162402149097

**Published:** 2016

**Authors:** Edie Benedito Caetano, Luiz Ângelo Vieira, Mauricio Ferreira Caetano, Cristina Schmitt Cavalheiro, Mauro Razuk, João José Sabongi

**Affiliations:** 1. Pontifícia Universidade Católica de São Paulo, Faculdade de Ciências Médicas e da Saúde, Campus Sorocaba, Sorocaba, SP, Brazil

**Keywords:** Arteriovenous anastomosis, Nervous system malformations, Median nerve, Ulnar nerve

## Abstract

**Objective:**

: This paper reports the incidence, origin, course and anatomical relationships of intramuscular Martin-Gruber anastomosis.

**Methods:**

: Anatomical dissection of 100 limbs from 50 adults cadavers was performed. The intramuscular Martin-Gruber anastomosis was found in five forearms, three in the right and two in the left side, one was bilateral. All communication were located between the anterior interosseous nerve and the ulnar nerve.

**Conclusion:**

: The purpose of intramuscular Martin-Gruber anastomosis, which we found in 5% of dissected limbs, is to supply the flexor digitorum profundus muscle and it is unlikely to have any influence on the innervation of the intrinsic muscles of the hand. Level of Evidence IV, Cases Series.

## INTRODUCTION

The nerve communication between the median and ulnar nerves is an anatomical variation that can occur in different locations in the upper limb. The nerve communication between the median and ulnar nerves may occur in the forearm ("Martin-Gruber" anastomosis), between the thenar motor branch of the median nerve and the deep motor branch of the ulnar nerve in the palm of the hand ("Cannieu and Riché" anastomosis), between the sensory branches of both nerves, also in the palm of the hand ("Berretini" anastomosis). Anatomical and electrophysiological studies suggest that these communications have important clinical and surgical implications. Several case reports on isolated injuries of the median and ulnar nerves showed differences from the classic pattern of innervation of these muscles suggested by anatomy treaties. The knowledge of anatomical variations in the innervation of these muscles is important for diagnosis and treatment of nerve damage and compression syndromes.

The Swedish anatomist Martin^1^, in 1763, was the first to consider the possibility of a connection between the fascicles of the median and ulnar nerves in the forearm. In the following century, in 1870, Gruber^2^ dissected 250 forearms and found 38 nerve connections. Since then, this neural communication is known as Martin-Gruber anastomosis.

The incidence of Martin-Gruber anastomosis was described by Gruber^2^ as (15.2%), Thomson^3^ (15.5%), Kimura et al.^4 ^(17%), Uchida e Sugioka^5^ (17%), Amoiridis^6^ (32%), Nakashima^7^ (21.3%), Shu et al.^8^ (23.6%), Rodriguez-Niedenfuhr^9^ (13.6%), Erdem et al.^10^ (27%), Sarikcioglu et al.,^11^ Prates et al.^12^ (7.8%), Lee et al.^13^ (39%), Kazaros et al.^14^ (10%),

Almeida et al.,^15^ and Felippe et al.^16^ (10%). Most of these authors consider that this anastomosis involves axons leaving the main trunk of the median nerve or anterior interosseous nerve, crossing the forearm to join the main trunk of the ulnar nerve, causing changes in the innervation of the intrinsic muscles of the hand. However, the existence of intramuscular anastomosis was only reported by Verchere,^17^ Nakashima,^7^ and Rodriguez-Niedenfuhr.^9^

In this study we report the Martin-Gruber anastomosis in 27 limbs. Of these connections, five occurred within the muscle mass of the deep flexor digitorum (intramuscular anastomosis). This article presents exclusively the anatomical details with the intramuscular connection resulting from these dissections.

## MATERIALS AND METHODS

One hundred forearms of 50 adult cadavers from the Anatomy discipline, *Faculdade de Ciencias Médidas e da Saúde da Pontíficia Universidade Católica de São Paulo* (Sorocaba campus), SP, Brazil, were dissected to perform this study. Forty six cadavers were male and four were female. The age ranged from 28 to 77 years old, 27 were white and 23 non-white. The pieces were previously prepared with 10% formaldehyde and glycerine solution. Forearms deformed by trauma and malformations were excluded from the sample.

The dissection was performed through a midline incision around the forearm and a lower third of the arm, two flaps including skin and subcutaneous tissue were folded to the radial and ulnar sides, respectively, and the same was done for the forearm fascia exposing, thus, all muscles.

All muscles of the forearm were dissected; innervation and the presence of nerve communication between the nerves of the forearm was analyzed. All anatomical variations found were recorded, noted and photographed. A Keeler 2.5X magnifying glass (Germany) was used for magnification. Besides investigation of "Martin-Gruber" nerve communication, the relation of Gantzer muscle with the anterior interosseous nerve and the median nerve, as well as the anatomical variations of the forearm muscles were analyzed. This study was approved by the Ethics Committee of *Faculdade de Ciências Médicas e da Saúde, Pontíficia Universidade Católica de São Paulo* (CAAE n° 43267715.2.0000.5373).

## RESULTS

We observed Martin-Gruber anastomosis in 27 of 100 forearms dissected, and in five limbs the nerve connection was reported inside the muscle mass of the deep flexor digitorum (intramuscular anastomosis). Regarding topographical situation, intramuscular communications occurred in the proximal third of the forearm, three on the right antimere and two on the left, and one of these was bilateral.

**Figure 1.**
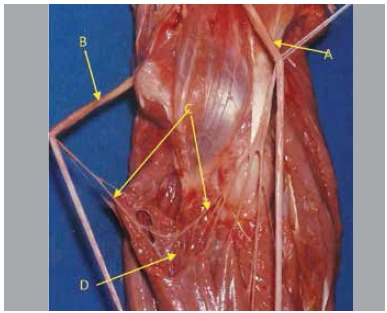
(A) Median nerve; (B) Ulnar nerve; (C) Intramuscular Martin-Gruber Anastomosis; (D) Flexor digitorum profundus muscle.

**Figure 2.**
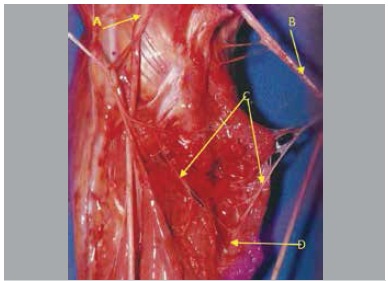
(A) Median nerve; (B) Ulnar nerve; (C) Intramuscular Martin-Gruber Anastomosis; (D) Flexor digitorum profundus muscle.

**Figure 3.**
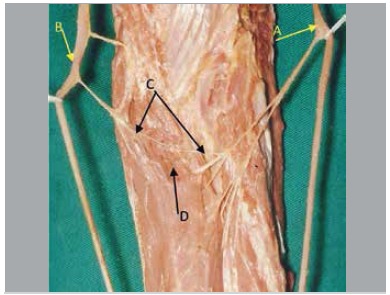
(A) Median nerve; (B) Ulnar nerve; (C) Intramuscular Martin-Gruber Anastomosis; (D) Flexor digitorum profundus muscle.

**Figure 4.**
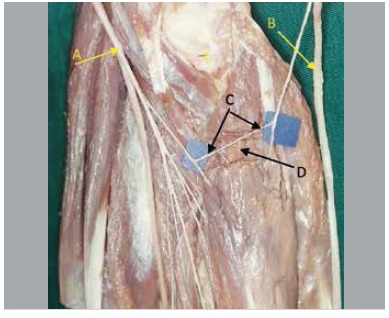
(A) Median nerve; (B) Ulnar nerve; (C) Intramuscular Martin-Gruber Anastomosis; (D) Flexor digitorum profundus muscle.

**Figure 5.**
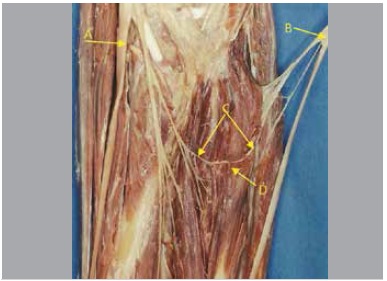
(A) Median nerve; (B) Ulnar nerve; (C) Intramuscular Martin-Gruber Anastomosis; (D) Flexor digitorum profundus muscle.

In five pieces nerve fascicles originated from the anterior interosseous nerve in a variable location at distal direction, variation of obliquity, posterior to the ulnar artery, penetrating the muscle mass of the deep flexor digitorum, communicating with the ulnar nerve inside the muscle. We found that from this nerve connection there were fascicles directed to the deep flexor digitorum muscle. (Figures 1-5) 

## DISCUSSION

The clinical implication of classical Martin-Gruber anastomosis is to enable the transfer of nerve fascicles between the median and ulnar nerves and, thereby, to alter the normal pattern of innervation of the intrinsic muscles of the hand. Thomson,^3^ Kimura et al.,^4^ Uchida e Sugioka,^5^ Shu et al.,^8^ Rodriguez-Niedenfuhr,^9^ Sarikcioglu et al.,^11^ Lee et al.,^13^ Kazaros et al.,^14^ and Felippe et al.^16^ considered that this anastomosis involves axons leaving the main trunk of the median nerve, or anterior interosseous nerve, crossing the forearm to join the main trunk of the ulnar nerve, causing innervation changes in the intrinsic hand muscles.

Martin-Gruber anastomosis has significant clinical importance for understanding certain injuries of the median and ulnar nerves and compression syndromes. Two cases described thereafter demonstrate this importance. Sraj et al.^18^ reported the case of a patient who had every symptoms of carpal tunnel syndrome, however, provocative Tinel signal test and Phalen test were negative. The patient presented obvious signs of ulnar nerve compression at the elbow. The nervous stimulus at the epitrochlea-olecranon groove triggered the typical symptoms of carpal tunnel syndrome, which indicates the transfer of sensitive (afferent) nerve fascicles of the ulnar nerve to the median nerve. Streib 19 reported the case of a 77 year-old patient complaining of hand weakness. Electrical stimulation has demonstrated that the response of the muscles in the thenar region had amplitude greater than 50% when the median nerve was stimulated in the wrist in relation to the elbow. The opposite occurred regarding the ulnar nerve, amplitude was 50% higher in the elbow. There is no doubt that in this case nerve communication occurred at the forearm (Martin-Gruber anastomosis).

We classified these 27 nerve connections in six types. In five of them (total of 22 pieces), we observed that these communications could alter the normal pattern of innervation of the intrinsic muscles. However, it is very unlikely that the changes in the intrinsic hand muscles may occur in intramuscular anastomoses, which purpose, in our interpretation, was exclusively to innervate the flexor digitorum profundus muscle.

The existence of the intramuscular anastomosis was only mentioned by Verchere^17^ and Nakashima,^7^ the latter reported the intramuscular connection in six of 30 dissected cases (20%), and this author believed that these nerve fascicles were destined to the flexor digitorum profundus muscle. Almeida et al.,^15^ analyzing the type of anastomotic presentation, found that two of five anastomoses originated from the muscular branches of the flexor digitorum profundus muscle, but did not inform whether these communications have occurred inside the muscle mass. Thomson,^3^ Lee et al.^13^ and Piagkou et al.^20^ mentioned, while ranking Martin-Gruber anastomosis, that these nerve connections can only innervate the flexor digitorum profundus muscle, but did not mention that this communication takes place inside the muscle mass. Rodriguez-Niedenfuhr^9^ described in details the intramuscular connection stating that its presence is extremely rare because it was recorded only in 1.3% of 236 dissected limbs and reported that intramuscular connection was represented by a single branch that originated from the anterior interosseous nerve, penetrated the muscle mass of the flexor digitorum profundus muscle without providing any nerve contribution to this muscle, and communicated with the ulnar nerve. They report that the intramuscular course of the nerve communication can be a potential nerve compression site which would be a clinical implication of this connection. Our findings agree with those of Nakashima,^7^ however differ completely from the description of Rodriguez-Niedenfuhr,^9^ because we consider that the purpose of intramuscular communication was to innervate the flexor digitorum profundus muscle, as in the five cases we reported with enough evidence, penetration of muscle mass fascicles of the flexor digitorum profundus muscle. 

## CONCLUSION

The knowledge of anatomical variations regarding hand innervation has a significant importance, particularly when considering physical examination, prognosis, diagnosis and surgical treatment. If these variations are not valued, mistakes and consequences are inevitable. We believe, however, that intramuscular nerve communications, reported in 5% of 100 members dissected, are intended only to innervate the flexor digitorum profundus muscle and it is unlikely to have any influence on the innervation of intrinsic muscles of the hand.
